# Controlled Release of 5-FU from Chi–DHA Nanoparticles Synthetized with Ionic Gelation Technique: Evaluation of Release Profile Kinetics and Cytotoxicity Effect

**DOI:** 10.3390/jfb11030048

**Published:** 2020-07-08

**Authors:** Mariarosa Ruffo, Ortensia Ilaria Parisi, Francesco Patitucci, Marco Dattilo, Rocco Malivindi, Fabio Amone, Catia Morelli, Alessandra Nigro, Diego Sisci, Francesco Puoci

**Affiliations:** 1 Department of Pharmacy, Health and Nutritional Sciences, University of Calabria, 87036 Rende, Italy; mariarosa.ruffo@unical.it (M.R.); ortensiailaria.parisi@unical.it (O.I.P.); fra.pati@hotmail.it (F.P.); marco.dattilo@unical.it (M.D.); rocco.malivindi@unical.it (R.M.); catia.morelli@unical.it (C.M.); nigroale16@gmail.com (A.N.); dsisci@unical.it (D.S.); 2 Macrofarm s.r.l., c/o Department of Pharmacy, Health and Nutrition Sciences, University of Calabria, 87036 Rende, Italy; amonefabio@gmail.com

**Keywords:** Chi-DHAr nanoparticles, drug delivery, 5-FU, release profile, cytotoxicity studies

## Abstract

The ionic gelation technique allows us to obtain nanoparticles able to function as carriers for hydrophobic anticancer drugs, such as 5-fluoruracil (5-FU). In this study, reticulated chitosan– docosahexaenoic acid (Chi–DHAr) nanoparticles were synthesized by using a chemical reaction between amine groups of chitosan (Chi) and carboxylic acids of docosahexaenoic acid (DHA) and the presence of a link between Chi and DHA was confirmed by FT-IR, while the size and morphology of the obtained Chi-DHAr nanoparticles was evaluated with dynamic light scattering (DLS) and scanning electron microscopy (SEM), respectively. Drug-loading content (DLC) and drug-loading efficiency (DLE) of 5-FU in Chi-DHAr nanoparticles were 33.74 ± 0.19% and 7.9 ± 0.26%, respectively, while in the non-functionalized nanoparticles (Chir + 5FU), DLC, and DLE were in the ranges of 23.73 ± 0.14%, 5.62%, and 0.23%, respectively. The in vitro release profile, performed in phosphate buffer saline (PBS, pH 7.4) at 37 °C, indicated that the synthetized Chi–DHAr nanoparticles provided a sustained release of 5-FU. Based on the obtained regression coefficient value (R^2^), the first order kinetic model provided the best fit for both Chir and Chi-DHAr nanoparticles. Finally, cytotoxicity studies of chitosan, 5-FU, Chir, Chir + 5-FU, Chi-DHAr, and Chi-DHAr + 5-FU nanoparticles were conducted. Overall, Chi-DHAr nanoparticles proved to be much more biocompatible than Chir nanoparticles while retaining the ability to release the drug with high efficiency, especially towards specific types of cancerous cells.

## 1. Introduction

Chitin and chitosan are the second-most abundant naturally-occurring polysaccharides, following cellulose, and they can be easily extracted from wastes of the fishing industry. In particular, chitosan is a linear polysaccharide derived from *N*-deacetylation of chitin [1] and its structure, composed of alternating units of *N*-acetyl-d-glucosamine and d-glucosamine, makes it a versatile material as it has both amino and hydroxyl groups in the structure. Moreover, it has been widely used in the development of self-assembled polymeric nanoparticles since it is nontoxic, biocompatible, and biodegradable [2]. Nevertheless, its molecular weight, insolubility at physiological pH, and high viscosity may restrict its uses in vivo and, so the chitosan depolymerization products, like chitosan oligosaccharide with low-molecular weight (Chi), could be used in several fields because it could overcome these limitations. Due to its biological properties, Chi finds wide applications in the pharmaceutical and medical areas because of its ability to provide several self-assembled delivery systems [3], which are able to enhance the solubility of poorly water-soluble drugs [4]. Carbodiimide chemistry allows for the chemical reaction between amine groups of chitosan and carboxylic acids of several fatty acids such as oleic acid [5] and linoleic acid [6]. Two interesting modifications are *N*-alkylation and *N*-acylation, which can provide the addition of hydrophobic pendant groups on the Chi hydrophilic chain and change the hydrophilic/lipophilic balance of the polymer. The obtained amphiphilic Chi can self-assemble into nanoparticles which are able to encapsulate hydrophobic substances within the core. One of the most feasible reactions that can be carried out to achieve amphiphilic Chi involves the condensation of a fatty acid with the amino group of glucosamine monomer. These chemical modifications allow the chitosan structure to become amphiphilic and then for nanoparticles with a nanometric size to be obtained by using the ionic gelation technique. By using this method, it is possible to obtain nanoparticles with a regular surface due to the reaction between the primary amino groups of Chi and the negatively charged groups of tripolyphosphate (TPP), which is used as a crosslinking agent in this technique [7]. This ionic gelation technique allows the formation of a cross-linking between Chi and TPP, which avoids chemical cross linking because it is not only toxic to the organism but also can damage the drugs.

Docosahexaenoic acid (DHA) was used for the functionalization of Chi through a condensation reaction using *N*-(3-dimethylaminopropyl)-*N*′ ethylcarbodiimide (EDC) as coupling reagent. DHA is a natural product with a hydrophobic chain, and it belongs to the category of polyunsaturated fatty acids (PUFAs) and it is a *n*-3 polyunsaturated fatty acid with several health benefits such as having a hypotriglyceridemic, anti-inflammatory, anticancer, antioxidant and antidepressant effect. In addition, various studies indicate anti-inflammatory and insulin-sensitizing effects of this fatty acid in metabolic disorder [8]. The reaction between DHA and Chi allows it to encapsulate 5-fluoruracil (5-FU) that has poor solubility and, moreover, to obtain a drug delivery system with a better compatibility with biological systems [8].

5-FU is widely used in the treatment of several cancers such as colorectal, brain and breast cancer but, when the aim is to maintain its serum concentrations at high levels, a continuous administration is necessary [9]. On the other side, if 5-FU maintains high levels in serum concentrations, it is involved in severe toxic effects and, for this reason, a system for controlled release of 5-FU, such as nanoparticles, may overcome this problem. This kind of drug delivery system can reduce its adverse effect and enhance its therapeutic index [10].

The main purpose of this study was to develop conjugate Chi–DHA nanoparticles as a drug delivery system for 5-FU. The importance and the originality of this research study is ascribable to the use of DHA for the functionalization of Chi that allowed the development of a new drug delivery system able to encapsulate 5-FU that has poor solubility. The physicochemical characterization was performed by using Fourier transform infrared spectroscopy (FTIR) and by comparing the swelling ability of Chi and of Chi–DHA conjugate. Then, Chi–DHA conjugate was used for the preparation of Chi-DHAr + 5-Fu nanoparticles. The obtained nanoparticles were tested in terms of dimension and morphology by using dynamic light scattering (DLS) and TEM and they were further analyzed in terms of 5-FU loading content and efficiency. The aim was to evaluate the release profile of 5-FU from Chir and Chi-DHAr nanoparticles, zero order and first order kinetic models were used. Preliminary in vitro cytotoxicity studies were carried out on MCF-7 breast cancer cells and HeLa cervical cancer cells in order to assess the levels of cytotoxicity in the two cancer cell lines, as an indication of the potential of these synthesized Chi-DHAr + 5-Funanoparticles for future drug delivery applications.

## 2. Results and Discussion

### 2.1. Preparation of Chi-DHA Conjugate

**Figure 1:**
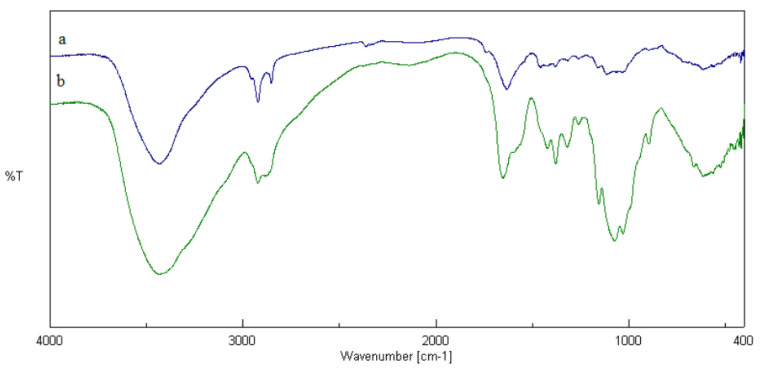
FT-IR spectra of (**a**) chitosan– docosahexaenoic acid (Chi-DHA) and (**b**) chitosan (Chi).

The aim was to modify the chitosan hydrophilic groups with hydrophobic compounds (such as fatty acids) and an EDC carbodiimide cross linker was used. The reaction between the carboxyl group of DHA and EDC allowed an amine reactive O-acylisourea intermediate to form. This reacted with the amine of chitosan to form a Chi-DHA conjugate. In this conjugate, the amine group of chitosan and the carboxyl group of DHA are joined by a stable amide bond [11]. To confirm the presence of Chi-DHA conjugate, FT-IR analysis was performed. In Figure 1a,b, FT-IR spectra of chitosan and Chi-DHA are shown. These measurements revealed chitosan and chitosan-DHA characteristic absorption peaks at 2924, 2854, 1464, and 1182 cm^−1^, which were assigned to the skeletal vibration of C–H bending. Moreover, the characteristic peaks at 1739 and 1590 cm^−1^ were attributed to the stretching of amide I and amide II groups. If the IR spectrum of Chi-DHA is compared to the IR spectrum of Chi (Figure 1a,b) it is possible to see a decrease of the vibrational band at 3000–3600 cm^−1^, which is related to a lower concentration of amino groups of chitosan after reaction with acids [3]. With the aim of verifying the increased hydrophobicity of Chi-DHA conjugate, swelling studies were performed, and the obtained data were compared to those obtained in Chi alone. The obtained results underline that the lowest swelling index was obtained in Chi-DHA conjugate, which may be related to the reduction of free hydrophilic groups in the conjugate. In fact, the swelling index of Chi alone, after dispersion in PBS pH 7.4 for 24 h, was about 127% ± 0.1% while, the swelling index of Chi-DHA conjugate was about 67% ± 0.2%.

### 2.2. Morphological Characterization of Chi-DHAr Nanoparticles

**Figure 2:**
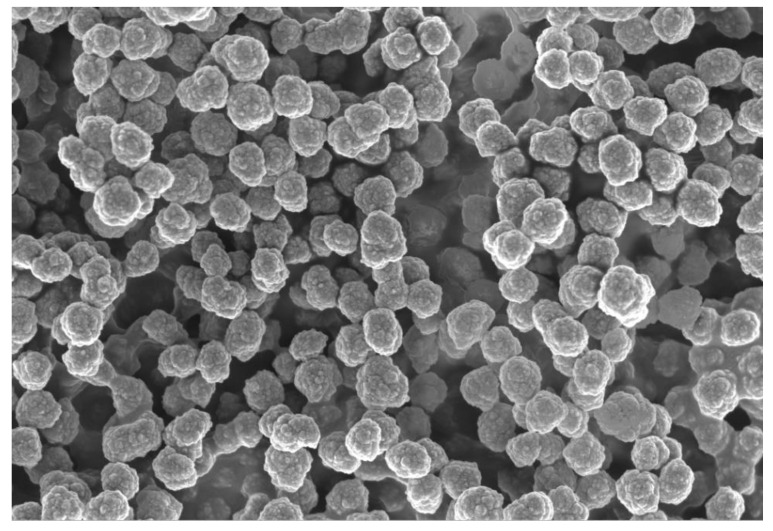
Scanning electron microscopy images of Chi-DHAr nanoparticles.

In this study, Chi-DHAr nanoparticles were synthetized using the ionic gelation method. This technique consists of ionic interaction between the negatively-charged polyanion of TPP and the positively-charged amino group of Chi, which makes possible the formation of nanoparticles through the formation of intra- and intermolecular cross-linkages [12]. To study the morphology and the dimension of Chi-DHAr nanoparticles, SEM and DLS instruments were used. The SEM analyses (Figure 2) revealed that the obtained nanoparticles had a spherical morphology with a regular surface and a dimension of 314 ± 0.019 nm. The size distribution of Chi-DHAr nanoparticles was also calculated by DLS analysis (Table 1). DLS measurements revealed that the dimension of the obtained nanoparticles was about 422 nm with a polydispersity index (PI) of 0.063. The value of PI is an index of the size distribution of nanoparticles and a value less than 0.1 indicates a homogeneous population of particles. The differences in size between DLS and SEM measurements were probably due to the different principles in the function of these instruments [13] and also to the different pretreatments that the samples undergo. Moreover, it is important to underline that SEM measurement is mostly used to verify nanoparticle shape and morphology, while DLS analysis is used as a prediction of the average size and polydispersity index of nanoparticles [14]. The DLS instrument was also used to evaluate the dimension of control nanoparticles (Chir nanoparticles) and the obtained data are reported in Table 1.

### 2.3. Drug Loading and Release

The DLC and DLE of Chir + 5-FU (non-functionalized nanoparticles) and of Chi-DHAr + 5-FU (functionalized nanoparticles) were calculated from Equations (1) and (2), respectively, using a UV spectrophotometer. Nanoparticles were left to react with a drug concentration of 5 mg/mL for 72 h in dark conditions and, at the end of the impregnation process, the concentration of unloaded 5-FU was measured by UV-VIS spectroscopy and the obtained results are reported in Table 2. The obtained values of DLC and DLE were probably due to the presence of DHA, which modified the structure of chitosan and improved the solubility of hydrophobic drugs [15].

At the end of the impregnation process, with the aim of comparing the size of particles before and after the addition of 5-FU, the dimension of the obtained nanoparticles was evaluated by using a DLS instrument (Table 3). The obtained results showed that the 5-FU loading process tended to increase the size of nanoparticles.

**Figure 3:**
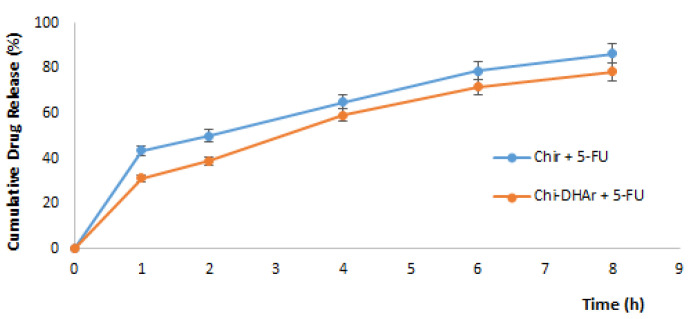
Cumulative release profiles of 5-FU from Chir and Chi-DHAr nanoparticles.

Drug release behavior of loaded 5-FU in Chir and Chi-DHAr nanoparticles was investigated in a phosphate buffer solution (pH 7.4) at 37 °C for 8 h. As shown in Figure 3, in the first hour 5-FU released from Chi-DHAr and from Chir was 31.3% and 43.4%, respectively. After 60 min, sustained release was also observed; the cumulative release of 5-FU increased slowly with time and at the end of 8 h, reached 86.5 and 78.5 for Chir + 5-FU and Chi-DHAr + 5-FU, respectively. The drug release results demonstrated that the in vitro 5-FU release from Chi-DHAr was much less if compared with drug release from Chir and this was probably due to the higher hydrophilicity in Chir nanoparticles. In agreement with the results of the present study, Zehu et al. found that the release of 5-FU from Chi nanoparticles and from N-PCCs27 (water-soluble biomimetic phosphorylcholine-bound chitosan) nanoparticles was similar, but in the N-PCCs27 sample the release of 5-FU was slightly higher, probably due to it improved hydrophilicity [16]. The obtained results suggest that the synthetized Chi-DHAr nanoparticles provided a sustained release of 5-FU, allowing the plasma drug concentration to be maintained and at the same time avoiding multiple drug dosing and the side effects of 5-FU. The higher hydrophobicity of synthetized Chi-DHAr nanoparticles allowed a sustained release of drug to be obtained, with a total amount of 5-FU lower than Chir nanoparticles. This result was in line with the aim of this study.

For further understand the release profile of 5-FU loaded in Chir and Chi-DHAr nanoparticles, zero-order (Equation (1)) and first-order (Equation (2)) kinetic models were used.

**Figure 4:**
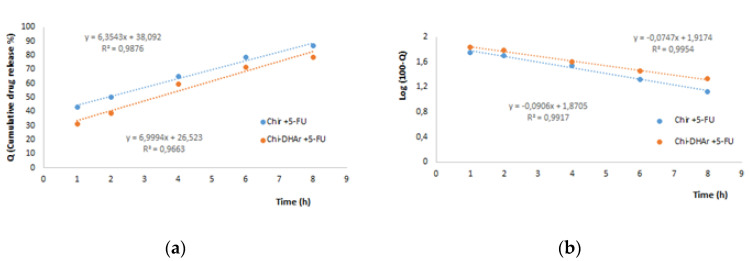
(**a**) Zero-order and (**b**) first-order release kinetic models for Chir + 5-FU and Chi-DHAr + 5-FU.

Q = K_0_t (1)

where Q is the amount of drug at time t and K_0_ represents the zero-order kinetic constant.

(2) $$\log Q=\log Q_{0}-\frac{K_{1}}{2.303}t$$

where Q is the amount of drug at time t, Q_0_ is the amount at time zero and K_1_ represents the first-order constant. The obtained data were reported as log of the cumulative of % drug remaining within the particles as a function of time. The zero and first release kinetic models are represented in Figure 4. In Table 4 it is possible to see the regression coefficient value (R^2^) obtained from the zero-order and first-order kinetic models, which were used to study the kind of release of 5-FU from Chir and Chi-DHAr nanoparticles, under the tested release conditions. As reported in Table 4, the first-order kinetic model provided the best fit for both Chir and Chi-DHAr nanoparticles due to the higher R^2^. However, as reported by Abouelmagd et al. [16], in this kind of release study, in which dialysis membranes are used, it could happen that the release profile of a poorly-water-soluble drug like 5-FU may be underestimated. This happens when the amount of nanoparticles used for the release studies is not sufficiently low and consequently the amount of drug that is released from nanoparticles could precipitate in the PBS medium or in the dialysis membranes. As a result of this, a low concentration of drug is found in the release medium and this data could be, consequently, interpreted as sustained drug release.

With the aim of overcoming this limitation and having a result that was a better fit with the release profile of 5-FU, low amounts of Chi-DHAr + 5-FU and Chir + 5-FU nanoparticles (10 mg) were used. Based on the obtained R^2^ values, which are reported in Table 4, the rate of 5-FU released, in both nanoparticles, better fitted the first-order kinetic model and so the release is dependent on drug concentrations.

### 2.4. Cytotoxicity Studies

**Figure 5:**
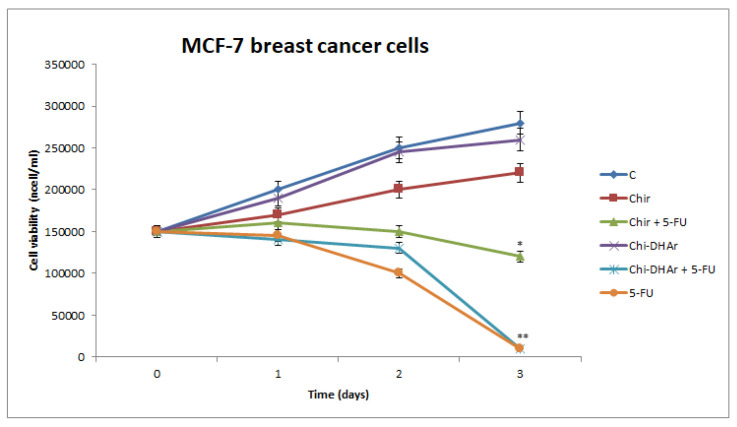
The effect of chitosan-based compounds on cell viability was determined in MCF-7 breast cancer cells at 1, 2, and 3 days. 5-FU was used as positive control. Values represent the mean of three independent experiments (* *p* < 0.05 Chir + 5-FU vs. untreated (C); ** *p* < 0.01 Chi-DHAr + 5-FU vs. Chir + 5-FU and vs. untreated (C)).

**Figure 6:**
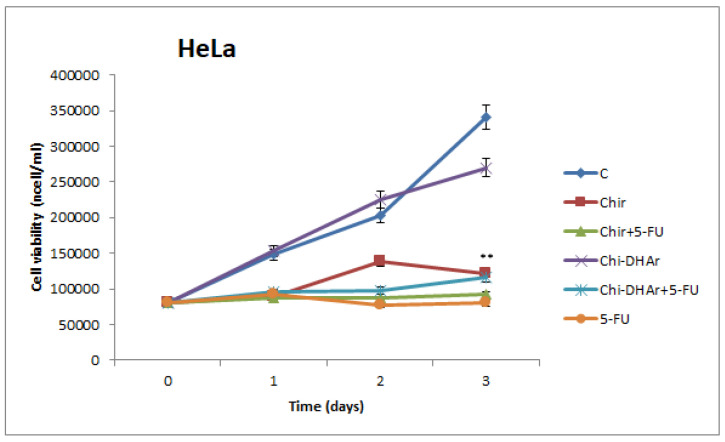
The effect of chitosan-based compounds on cell viability was determined in cancerous HeLa cells at 1, 2, and 3 days. 5-FU was used as positive control. Values represent the mean of three independent experiments (** *p* < 0.01 vs. untreated (C) and Chi-DHAr).

**Figure 7:**
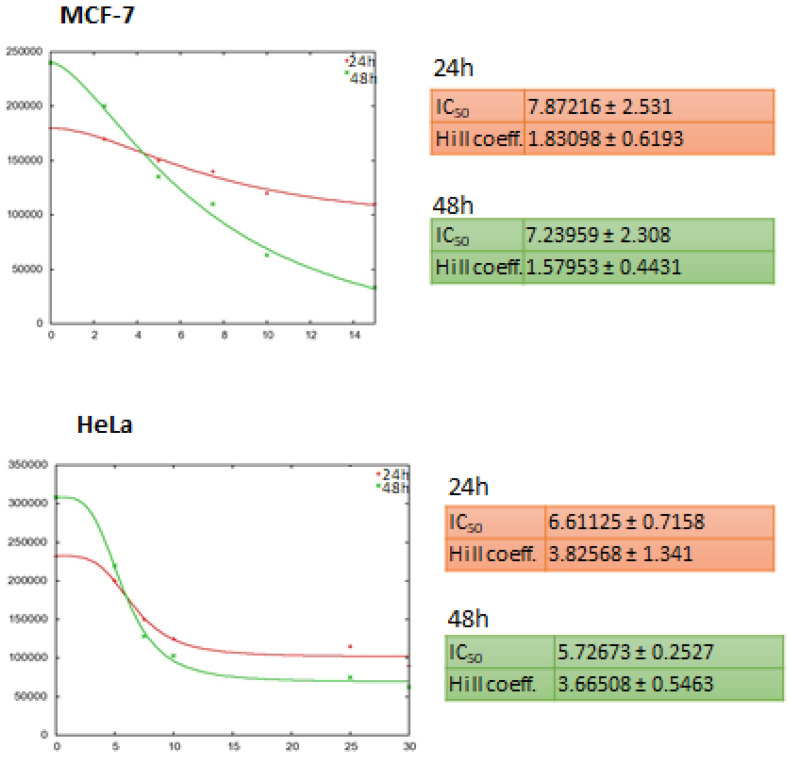
IC_50_ of 5-FU was calculated by means of the open source http://Ic50.tk, both for MCF-7 and HeLa cell lines. The concentration of 7.5 μg/well used for growth experiments derives from a median value between IC_50_ at 24 h and 48 h. The same concentration was suitable also for the HeLa cell line, based on the calculated IC_50_ value at 24 h.

The effect on cell viability of chitosan-based nanoparticles were assessed by trypan blue assay. Our results showed that in MCF-7 breast cancer cells Chi-DHAr nanoparticles not only proved to be more biocompatible than Chir nanoparticles, but they were also able to release drug more efficiently, in a trend that was similar to that of free 5-FU. Indeed, both Chir + 5-FU and Chi-DHAr + 5-FU caused a decrease in cell viability as soon as after two days of exposure, but Chi-DHAr + 5-FU was much more effective than Chir + 5-FU (Figure 4). As observed in MCF-7, Chi-DHAr is also not toxic for the HeLa cell line (Figure 5), while Chir nanoparticles revealed a cytotoxic activity, probably due to the absence of the linked DHA, which was comparable to that of 5-FU-bearing compounds and free 5-FU. Moreover, although the anticancer activity of Chi-DHAr + 5-FU was slightly lower than that of Chir + 5-FU, the therapeutic effect was still highly satisfactory. However, it is worth underlining that the growth inhibition exerted by Chir + 5-FU was very similar to that of Chir, evidencing a clear toxicity of the vehicle alone, independently of the presence of the antineoplastic drug. In Figure 6, representative images of HeLa cells, showing the effect of the different nanoparticles at 72 h of treatment are reported. In the Figure 7, IC_50_ both for HeLa and MCF-7 cell line is represented.

### 2.5. Cellular Uptake Studies of 5-FU

After 4 h of incubation, cells were lysed and the amount of 5-FU detected within the cells was analyzed by HPLC. The obtained results (Table 5) evidence that the amount of detected 5-FU in Chi-DHAr nanoparticles both in MCF-7 breast cancer cells and in HeLa cells was higher than detected 5-FU in Chi-DHAr nanoparticles. The results point to the key role of DHA in the process of intracellular uptake and underline its important role as a drug delivery system because it can reach the target site in the cell interior.

## 3. Materials and Methods

### 3.1. Chemicals

Chitosan (low molecular weight of 50–190 KDa), docosahexaenoic acid (DHA), 1-ethyl-3-(3-dimethylaminopropyl) carbodiimide (EDC), glutaraldehyde solution (25% *w*/*w*), mineral paraffin oil, sorbitan monoleate (SPAN 80^®^), 5-fluoruracil (5-FU), tripolyphosphate (TPP), and bovine serum albumin (BSA) were purchased from Sigma-Aldrich (Milan, Italy).

### 3.2. Cell Cultures

HeLa cervical adenocarcinoma cells and MCF-7 breast cancer cells were provided by the American Type Culture Collection (ATCC). MCF-7 cells were grown in Dulbecco’s Modified Eagle’s Medium/Nutrient Mixture F-12 Ham (DMEM/F12) plus 5% fetal bovine serum (FBS, Invitrogen); HeLa cells were grown in Modified Eagle’s Medium (MEM) with 10% FBS. All culture media were supplemented with 100 IU mL^−1^ penicillin, 100 mg mL^−1^ streptomycin, and 0.2 mM L-glutamine. All media, FBS, and reagents for cell culture were purchased from Life Technologies, Monza, Italy. Cells were maintained as monolayer cultures in a humidified incubator at 5% CO_2_ and 37 °C.

### 3.3. Instrumentation

All used solvents are reagent grade or HPLC-grade and so they were used without further purification. Dialysis membranes of 6–27/32” Medicell International LTD (MWCO: 12–14,000 Da) were provided by Spectrum Laboratories Inc., Dalton, U.S.A. IR spectra were recorded as KBr pellets on a Jasco FT-IR 4200 (Easton, MD, USA). Absorption spectra were recorded with a Jasco V-530 UV/Vis spectrometer (Easton, MD, USA). Particles size and distribution were determined by dynamic light scattering analyses using a 90 Plus particle size analyzer (Brookhaven Instruments Corporation, New York, NY USA), at 25.0 ± 0.1 °C by measuring the autocorrelation function at 90°. The polydispersity index was used as a measure of the width of size distribution. P.I. (Polydispersity Index) less than 0.1 indicates a homogenous population of particles. Scanning electron microscopy micrographs were obtained with a Jeol JSMT 300 A; samples were made conductive by gold layer deposition on particles surface in a vacuum chamber. For HPLC analysis of 5FU, a Jasco BIP-I pump and Jasco UVDEC-100-V detector set at 266 nm were used. A 250 × 4 mm C-18 Hibarw column, 10 mm particle size (Merck) was employed. The mobile phase was methanol/phosphate buffer solution (PBS) 5 mM, pH 6.8 (9/1, *v*/*v*) and the flow rate was 0.5 mL/min. The HPLC system used to carry out the HPLC analysis of cellular uptake of 5-FU was a Varian 900- LC Series Liquid Chromatograph equipped with a fluorescent detector. The mobile phase used in this analysis was a solution of methanol in water (70% *v*/*v*). The column effluent was monitored by fluorescence detection with excitation and emission wavelengths of 346 nm and 395 nm, respectively. The fluorescence intensity was measured by fluorescence spectrophotometer.

### 3.4. Preparation of Chi-DHA Conjugate

The formation of amide linkages between DHA and Chi was mediated by EDC, as described by Liu et al. [17] with slight modifications. Briefly, 500 mg of Chi were dissolved in 70 mL of 0.5% (*v*/*v*) aqueous acetic acid solution and left in agitation until complete dissolution to make the solution A. Then, 250 mg of DHA were dissolved in an EDC ethanol solution (75 mL, 2.1 mg mL^−1^) and after that, this solution was poured dropwise into solution A, maintaining the solution constantly under magnetic stirring. The final solution was stirred at 40 °C for 24 h. After achieving completeness of the reaction, the final product was dialyzed for 24 h against a water/ethanol (1:1) using a 12–14 kDa dialysis membrane. After the dialysis process Chi-DHA was frozen and freeze-dried to a powder. To better verify the coupling between Chi and DHA, its swelling ability was evaluated and the results were compared with those obtained with Chi alone. To do this, 50 mg of Chi-DHA conjugate and Chi were placed into a tared 5 mL sintered glass filter (Ø 10 mm; porosity, G3), following the protocol of Parisi et al. [18].

### 3.5. Synthesis of Chi-DHAr Nanoparticles

Chi-DHAr nanoparticles were synthetized with ionic gelation and oil in the water emulsion method. Chi-DHA conjugate (100 mg) was dissolved in 2.5 mL of a solution of acetic acid (1% *v*/*v*) and once the conjugate was dissolved, 20 mL of ethanol were added. Nanoparticles were then obtained by adding a solution of TPP (1 mg/mL in water) dropwise to the Chi-DHA solution in a ratio of 3:1 Chi-DHA:TPP. Finally, the solution was dialyzed and freeze dried to obtain Chi-DHAr nanoparticles [19].

### 3.6. Drug Loading and Release

Chi-DHAr nanoparticles were loaded with 5-FU according to the literature [20]. 80 mg of Chi-DHAr was added to a methanol solution of 5-FU (5 mg mL^−1^) and this was left to react for 72 h in dark conditions and under magnetic stirring. At the end of the impregnation process, the obtained particles were filtered and the excess of solvent was percolated at atmospheric pressure. The concentration of unloaded 5-FU was measured by UV-VIS spectroscopy through the calibration curve of 5FU in methanol at 264.5 nm. Finally, 5-FU-loaded particles were dried under vacuum overnight. The drug-loading content (DLC) and drug-loading efficiency (DLE) were calculated using Equations (3) and (4).

(3) $$\mathrm{DLC}(\%)=\frac{\mathrm{WLD}}{\mathrm{WLD}+\mathrm{WP}}\times 100$$

where WLD is the weight of loaded drug in nanoparticles and WP is the weight of polymer.

(4) $$\mathrm{DLE}(\%)=\frac{\mathrm{WLD}}{\mathrm{IWD}}\times 100$$

where IWD is initial weight of drug.

All the experiments were performed in three parallel studies.

In vitro 5-FU release from Chi-DHAr nanoparticles was studied by the dialysis bag diffusion technique [21]. The amount of 10 mg of 5-FU-loaded Chi-DHAr nanoparticles was transferred into the dialysis bag (Molecular Weight Cut-Off of 12–14 KDa) and then immersed into a vial containing 10 mL PBS at pH 7.4. The release study was started by maintaining the tested sample in constant agitation in a water bath at 37 °C for 8 h. Three millilitres of the outer solution was withdrawn at selected time intervals (1 h, 2 h, 4 h, 6 h, and 8 h) and then it was restored with 3 mL of fresh PBS. 5-FU concentration was quantified by HPLC analyses and the obtained percentages of released therapeutic agent were used to characterize the release profile. The measurements were repeated three times. The LOD (Limit of detection) value for 5-FU was 0.015 µg/mL, while LOQ (Limit of quantification) value was found to be 0.035 µg/mL.

### 3.7. Cell Viability

After seeding, cells were serum-starved with serum-free medium (SFM) overnight in order to synchronize their cell cycle and avoid differences in cell growth. The next day SFM was replaced by fresh medium plus 1% (*w*/*w*) FBS and 500 µg of nanoparticles (containing 1.5% of 5-FU, corresponding to 7.5 µg/mL of free drug) were added. Comparable amounts of polymer alone were used as a negative control, while 7.5 µg/mL of free drug was employed as positive control. After 1, 2, or 3 days, cells were harvested by trypsinization and incubated in a 0.5% trypan blue solution for 10 min at room temperature. Cell viability was determined by counting trypan-blue-negative cells in a Countess Automated Cell Counter (Life Technologies, Milan, Italy) [22].

### 3.8. Cellular Uptake Studies of 5-FU

The in vitro cellular uptake of drug from Chir + 5-FU and Chi-DHAr + 5-FU nanoparticles was evaluated by using HeLa and MCF-7 cancer cells. Briefly, cells were seeded in a 96-well microplate and incubated at 37 °C for 24 h. At the end of incubation time, the medium was replaced by serum- free medium containing 500 μg of nanoparticles containing 7.5 μg of free drug and these were incubated for further 4 h. After that cells were washed thrice with PBS followed by trypsinization. To obtain free 5-FU, the collected cells were lysed by sonication for 10 min and then 50 μL of (70% *v*/*v*) methanol in PBS (pH 7.4, 0.001 M) was added and cell lysates was obtained by centrifugation at 1500 rpm for 5 min and supernatant was collected for free 5-FU determination [23]. Drug uptake was evaluated by HPLC analysis. All the experiments were carried out in triplicate.

### 3.9. Statistical Analyses

Each measurement was carried out in three independent experiments, data are expressed as means (±SD) and were analyzed using one-way analysis of variance (ANOVA).

## 4. Conclusions

The aim of the present study was the development of Chi-DHAr nanoparticles for controlled release of 5-FU. Chi-DHAr nanoparticles were prepared by the ionic gelation technique, while Chi-DHA conjugate was prepared by using EDC carbodiimide cross linker. The obtained conjugate was investigated by FT-IR spectroscopy and swelling studies, which confirmed the presence of a stable amide bond between the carboxyl group of DHA and the amine group of Chi while the dimensions and surfaces of nanoparticles were investigated with DLS and SEM instruments. The obtained DLC and DLE values confirmed the good loading ability of Chi-DHAr nanoparticles if compared to the results obtained from Chir nanoparticles. Moreover, the in vitro release studies highlighted the 5-FU controlled-release behavior of Chi-DHAr nanoparticles that better fitted the first-order kinetic model. Finally, in vitro cytotoxicity studies showed how Chi-DHAr nanoparticles are less toxic than Chir only, but when conjugated to the antineoplastic drug, they retain a striking cytotoxic activity towards specific types of cancer cells (e.g., MCF-7). Therefore, further investigations are needed in order to identify other cell lines or tissues that might be preferentially targeted by Chi-DHAr nanoparticles based on specific molecular profiles and/or chemical–physical interaction between the nanoparticles and the cell membranes. The obtained data provides evidence that Chi-DHAr nanoparticles might represent a good vehicle for the controlled release of drugs, particularly 5-FU, to cancer cells. Moreover, for the first time, DHA was used for the functionalization of Chi and as a new drug delivery system able to encapsulate 5-FU that has poor solubility.

## Figures and Tables

**Table 1:** Dynamic light scattering measurements of Chi-DHAr and Chir nanoparticles.

Sample	Mean Diameter	Polydispersity Index
Chi-DHAr nanoparticles	421.8 ± 0.1 nm	0.063
Chir nanoparticles	402 ± 0.1 nm	0.081

**Table 2:** The percentages of drug-loading efficiency (DLE) and drug-loading content (DLC) of Chir + 5-FU and Chi-DHAr + 5-fluoruracil (5-FU) nanoparticles.

Sample	DLE (%)	DLC (%)
Chir + 5-FU	27.73 ± 0.14	5.62 ± 0.23
Chi-DHAr + 5-FU	33.74 ± 0.19	7.9 ± 0.26

**Table 3:** Dynamic light scattering measurements of Chir + 5-FU and Chi-DHAr + 5-FU nanoparticles.

Sample	Mean Diameter	Polydispersity Index
Chir + 5-FU	421 ± 0.3	0.08
Chi-DHAr + 5-FU	442 ± 0.2	0.07

**Table 4:** Release kinetics data for 5-FU-loaded Chir and Chi-DHAr nanoparticles.

Sample	Zero Order (r^2^)	First Order (r^2^)
Chir + 5-FU	0.9876	0.9917
Chi-DHAr + 5-FU	0.9663	0.9954

**Table 5:** Intracellular amount of 5-FU accumulated in MCF-7 and HeLa cell lines after 4 h of exposure to Chi-DHAr and Chir nanoparticles.

Sample	Amount of 5-FU in MCF-7 Cells (μg)	Amount of 5-FU in HeLa Cells (μg)
Chi-DHAr + 5-FU nanoparticles	5.4 ± 0.36	6.2 ± 0.2
Chir + 5-FU nanoparticles	3.5 ± 0.36	3.9 ± 0.3
